# Effect of Denture Tooth Material on Load Transmission Under Denture Bases

**Published:** 2018-09

**Authors:** Ramin Mosharraf, Farzad Ziaei, Mahsa Abbasi

**Affiliations:** 1 Professor, Dental Materials Research Center, Department of Prosthodontics, School of Dentistry, Isfahan University of Medical Sciences, Isfahan, Iran; 2 Graduate, Dental Students, Research Committee, School of Dentistry, Isfahan University of Medical Sciences, Isfahan, Iran; 3 Assistant Professor, Department of Prosthodontics, School of Dentistry, Shahrekord University of Medical Sciences, Shahrekord, Iran

**Keywords:** Denture Bases, Occlusal Forces, Acrylic Resins, Pressure, Artificial Tooth

## Abstract

**Objectives:**

Pressure transmission under denture bases can vary depending on the denture tooth material. The aim of the present study was to evaluate pressure transmission under denture bases using denture teeth of different materials in direct and indirect tooth contacts.

**Materials and Methods:**

In this in-vitro study, the pressure transmission generated by five types of denture teeth, including ceramic, nanocomposite, composite-acrylic resin, cross-linked acrylic resin, and polymethyl methacrylate (PMMA), under direct and indirect pressures was evaluated (n=10). The maximum pressure (MPa) was measured using a strain gauge. Data were statistically analyzed with one-way analysis of variance (ANOVA; α=0.05).

**Results:**

The denture tooth material had a significant effect on pressure transmission under denture bases (P<0.001). Under direct load, ceramic and PMMA teeth exhibited the maximum and minimum pressures, respectively, contrary to indirect load (P<0.001).

**Conclusions:**

Pressure transmission under denture bases significantly varies with the use of different denture tooth materials. Acrylic teeth could be the most favorable choice to reduce the pressure beneath denture bases. Nanocomposite and acrylic resin-composite teeth may be used as alternatives.

## INTRODUCTION

One of the major factors involved in residual ridge resorption in edentulous patients is the pressure applied by a denture. The process of residual ridge resorption is "chronic, progressive, and irreversible" [1]. There is variation in the magnitude and the pattern of the resorption [2]. Previous studies have shown that bone resorption happens when a high pressure is applied [3,4]. An increase in residual ridge resorption in denture wearers has been correlated with the pressure exerted by dentures [5]. However, it has been suggested that a proper amount of pressure (within the limits of physiological tolerance) could stimulate bone apposition. If the denture base prevents blood flow in the bone or induces inflammation in the mucoperiosteum, bone resorption may occur [6].

Fabrication of dental prostheses should be based on reducing the amount of load applied to the residual ridges. The selection of denture tooth materials is one of the important steps in the clinical settings of denture fabrication for reducing the pressure. Appropriate artificial denture teeth can resist pressure and prevent stress concentration in the underlying tissues [7]. The question is whether a material with a higher coefficient of elasticity, such as acrylic resin, would be less harmful to the residual ridges. Few studies have evaluated the pressure under the denture base when a substance is placed between the teeth [7–9].

Previous studies have reported that acrylic resin teeth have a good impact resistance and shock absorbability, whereas ceramic denture teeth have the maximum impact values [8,9].

Novel denture teeth have highly cross-linked occlusal and incisal surfaces to resist wear, and uncross-linked necks to allow for good chemical bonding to an acrylic denture base [10–12].

Phunthikaphadr et al [7] observed maximum pressure transmission with ceramic, nanocomposite, and acrylic denture teeth, respectively. The pressure values associated with ceramic denture teeth were significantly higher than those in other groups (P<0.001). Furthermore, they showed that nanocomposite denture teeth exhibited the lowest pressure transmission [7]. Nanocomposites have been introduced as a material for artificial denture teeth [13].

It has been reported that the interfacial surfaces between the polymer and nanoparticles are effective in absorbing stresses [14]. Arksornnukit et al [15] did not find any statistically significant differences in the average pressure transmission among different types of materials in 0-degree denture teeth, whereas they revealed the highest average pressure with 35-degree ceramic denture teeth, followed by 33-degree acrylic resin and microfilled composite resin denture teeth. The increased wear resistance of highly cross-linked acrylic resin teeth and composite resin teeth, compared to conventional acrylic resin teeth, has been reported by previous studies [12,16,17]. However, some in-vitro studies reported no significant differences between improved and conventional acrylic resin teeth in terms of the wear resistance [13,18,19].

Various techniques and measuring devices are available to record the pressure beneath the denture base, but strain gauge pressure transducers are most commonly used for measuring pressure at specific sites [20,21].

The coefficient of elasticity or the measure of elasticity is "the ability of a body to resist a distorting influence or deforming force and to return to its original size and shape when that influence or force is removed" [22]. The aim of the present study was to evaluate pressure transmission using different artificial denture tooth materials, including ceramic, nanocomposite, composite-acrylic resin, cross-linked acrylic resin, and PMMA, in direct and indirect contacts. The null hypothesis was that there would be no significant differences in pressure transmission among different artificial denture tooth materials.

## MATERIALS AND METHODS

Fifty pairs of mandibular and maxillary first molars made of five different types of denture tooth materials, including acrylic resin (AR); composite-acrylic resin (C-AR), nanocomposite (NC), ceramic (CR), and denture base resin (DBR; as the control group), were examined in this analytical cross-sectional study. The materials used in the present study are described in Table 1.

**Table 1.** Materials used in the study

** Group **	** Name and Composition **	** Manufacturer **	** City, Country **	** Mold Size **	** Mold Shade **
** AR **	Super-Newclar (acrylic resin with 7% cross-linking)	Ideal Makoo Co.	Tehran, Iran	N3	A3
** C-AR **	Super-Brilian (composite-acrylic resin)	Ideal Makoo Co.	Tehran, Iran	N3	A3
** NC **	B-star (nanocomposite)	Ideal Makoo Co.	Tehran, Iran	N3	A3
** CR **	Global Hawk nano steel teeth (ceramic teeth)	Caiyu Dental Materials Factory	Huizhou, China	30	A3
** DBR ** (Control group)	Acrylic resin denture base materia	Meliodent, Bayer Dental	Newbury, UK	-	-

AR=Acrylic Resin, C-AR=Composite-Acrylic Resin, NC=Nanocomposite, CR=Ceramic, DBR=Denture Base Resin

### Mounting of the specimens:

For the first four groups, 40 models were made of a denture tooth mounted in a cylinder-shaped acrylic baseplate resin base (Meliodent, Bayer Dental, Newbury, Berkshire, UK).

**Fig. 1:**
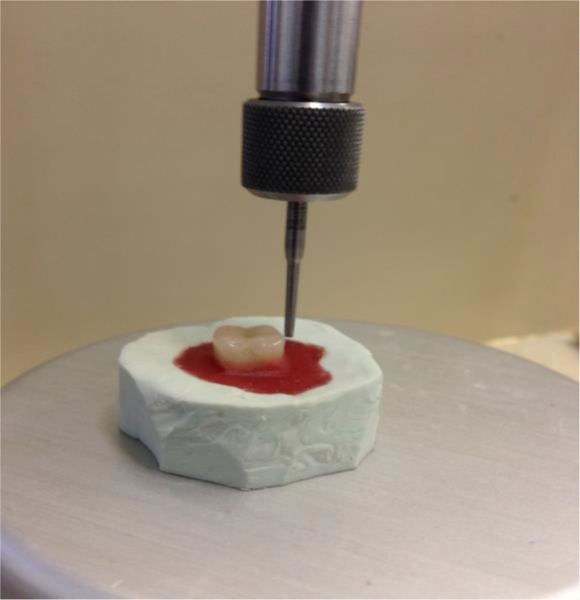
A dental surveyor was used to place each denture tooth on the surface of the wax perpendicular to the occlusal surface

For making each model, some melted baseplate wax (Modeling Wax; Dentsply DeTrey, Weybridge, Surrey, UK) was poured into a cylinder-shaped silicone mold (Speedex, Coltène/Whaledent AG, Altstätten, Switzerland), and a dental surveyor (Ney® Surveyor Parallometer System; Dentsply Ceramco, Burlington, NJ, USA) was used to place each denture tooth on the surface of the wax perpendicular to the occlusal surface (Fig. 1). All denture teeth were selected with the same mold size and shape as possible.

For making the specimens of the fifth group (DBR), a mold made of a putty-type silicone impression material (Speedex, Coltène/Whaledent AG, Altstätten, Switzerland) was used to duplicate and fabricate acrylic denture teeth from acrylic denture base resin. In all five groups, the same method was used for preparing each pair of maxillary and mandibular denture teeth.

The specimens were invested (PM Investment Material, VITA Zahnfabrik, Bad Säckingen, Germany) in denture flasks, and the acrylic baseplate resin packing procedure was carried out using a heat-polymerizing acrylic baseplate resin (Meliodent, Bayer Dental, Newbury, Berkshire, UK) in a conventional manner. After de-flasking, each specimen was adjusted to make a cylinder measuring 20 mm in diameter and 3 mm in thickness.

### The chewing simulator machine:

**Fig. 2:**
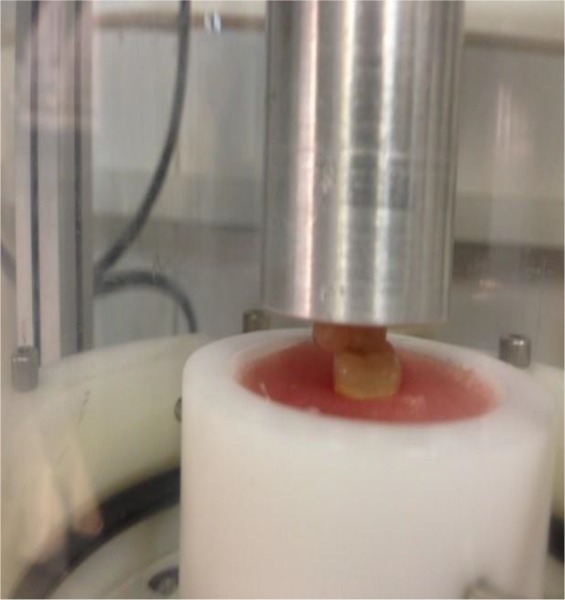
A specimen in the chewing simulator machine

After preparing all the specimens, each pair of maxillary and mandibular denture teeth was arranged in Class I occlusal relationship in a chewing simulator machine (Chewing Simulator CS-4.2, SD Mechatronic, Feldkirchen, Westerham, Germany; Fig. 2).

To achieve an ideal and uniform contact, occlusal adjustment with selective grinding method was carried out using a carbide bur (Abbott-Robinson HP Burs; Buffalo Dental Mfg. Co., NY, USA). The final polishing procedure was carried out using finishing discs (Bisco Inc., Schaumburg, IL, USA). A strain gauge sensor, designed and fabricated at Isfahan University of Technology, Isfahan, Iran, was placed under the first mandibular tooth at the center of the acrylic baseplate resin base and was calibrated by applying different values of force (N). Similar to a previous study by Arksornnukit et al [15], a 50 N load was used in the present study. In the first stage, the teeth were impacted together with a load of 50 N for 2 seconds at a crosshead speed of 1 mm/second, and the strain gauge sensor recorded the maximum pressure (MPa) as "the direct value". In the next stage, carrot cubes (10 mm^3^) were placed between the teeth, and the teeth were moved towards each other with a load of 50 N until they were at least 1 mm apart. The sensor recorded the maximum pressure value as "the indirect value". This process was repeated 10 times for each pair separately at a crosshead speed of 1 mm/second.

### Data analysis:

SPSS 16 software program (SPSS Inc., Chicago, IL, USA) was used for statistical analysis. Data were analyzed with one-way analysis of variance (ANOVA) and post-hoc Tukey's test (α=0.05). Pearson correlation coefficient was calculated to assess the correlation between direct and indirect loads.

## RESULTS

The means, standard deviations (SD), and standard errors (SE) of maximum pressure transmission in direct 2-body and indirect 3-body contacts for each specimen are shown in Table 2.

**Table 2.** Means and standard deviations (SD) of maximum pressure (MPa) transmission in direct 2-body and indirect 3-body contacts, and the results of the comparisons made using post-hoc Tukey's test

** Denture Tooth Material **	** Number **	** Direct Pressure Mean(±SD) **	** Indirect Pressure Mean(±SD) **
** CR **	10	5.2280(±0.15017) ^ a ^	1.6310(±0.12723) ^ A ^
** NC **	10	4.6320(±0.06443) ^ b ^	2.0420(±0.10075) ^ B ^
** C-AR **	10	4.4940(±0.09845) ^ b,c ^	2.2120(±0.10902) ^ C ^
** AR **	10	4.253(±0.1542) ^ d ^	1.948(±0.07421) ^ B,D ^
** DBR **	10	3.808(±0.12444) ^ e ^	2.446(±0.0782) ^ E ^

AR=Acrylic Resin, C-AR=Composite-Acrylic Resin, NC=Nanocomposite, CR=Ceramic, DBR=Denture Base Resin;

* Different letters show a significant difference between the groups (P<0.05)

In both direct 2-body and indirect 3-body contacts, one-way ANOVA revealed significant differences in maximum pressure transmission among different materials (P<0.001; Table 3). Therefore, the denture tooth material had a significant effect on pressure transmission under the denture base.

**Table 3.** One-way analysis of variance (ANOVA) for direct and indirect pressure transmission with different materials

		** Sum of Squares **	** df **	** Mean Square **	** F Distribution **	** Sig. **
** Direct Pressure **	Intergroup	10.859	4	2.715	179.403	P< 0.001
	Intragroup	.681	45	.015		
	Total	11.540	49			
** Indirect Pressure **	Intergroup	3.689	4	.922	92.516	P< 0.001
	Intragroup	.449	45	.010		
	Total	4.138	49			

df=degree of freedom

**Fig. 3:**
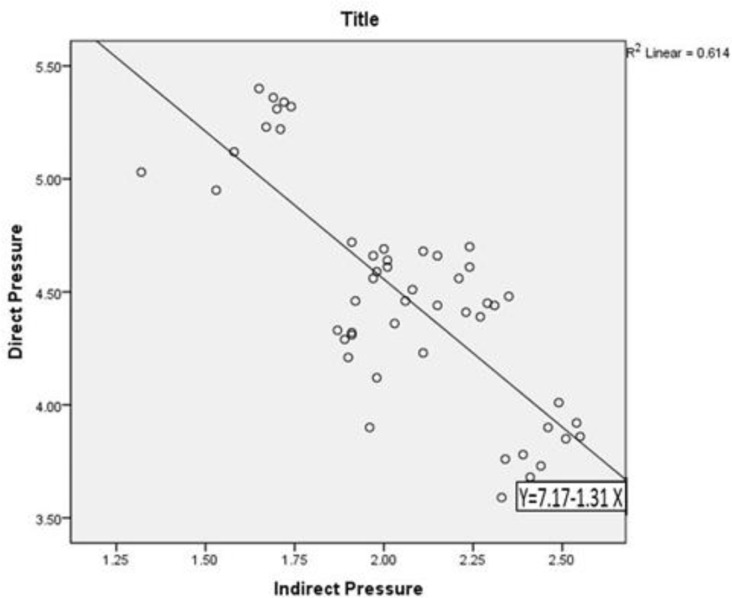
Spot diagram showing linear correlation between direct 2-body and indirect 3-body tooth contacts. Direct and indirect pressures are in MPa

Post-hoc Tukey's test revealed significant differences among the groups (P<0.001), except between NC and C-AR groups under direct load and between NC and AR groups under indirect load (Table 2). The correlation coefficient between direct 2-body and indirect 3-body tooth contacts was −.784 (R^2^ linear=0.618; Fig. 3). Therefore, under direct load, ceramic and PMMA teeth exhibited the maximum and minimum pressures, respectively, contrary to indirect load (P<0.001).

## DISCUSSION

In this study, there were significant differences in load transmission values with denture teeth made of different materials in two types of contact (direct 2-body and indirect 3-body). Therefore, the null hypothesis was rejected. This means that a suitable tooth material should be selected for the fabrication of complete dentures, especially when the pressure transmitted to the underlying tissues is a concern, e.g. in patients with higher force factors or with severe ridge resorption. The present study showed that ceramic teeth (CR) transmitted the maximum and minimum pressures in direct 2-body and indirect 3-body contacts, respectively. In contrast, acrylic teeth (AR) exhibited the maximum and minimum pressure transmission in indirect 3-body and direct 2-body contacts, respectively. Given this fact and by considering the negative coefficient of correlation between direct and indirect loads, it can be concluded that whenever the pressure transmission by teeth under direct load is increased, chewing the third body becomes more comfortable, and the force transmission under the denture base would decrease.

On the other hand, whenever the pressure transmission by teeth under direct load is decreased, chewing the third body becomes harder, and the force transmission under the denture base would increase. Thus, tooth selection must be based on a balance between these two extremes. In this study, three groups of Super-Newclar (AR), Super-Brilian (C-AR), and B-star (NC) had no significant differences in indirect 3-body contact, but since Super-Newclar (AR) group transferred significantly less pressure in direct 2-body contact, it can be concluded that this type of tooth provides the most proper balance between forces compared to other teeth.

Kawano et al [9] reported that the pressure induced by ceramic teeth with a PMMA resin base may be less than that induced by ceramic teeth alone since there is no chemical bond between ceramic teeth and PMMA resin base, and the transmitted force may be broken at the interface. It seems that the results of the present study confirm the fact that sufficient thickness of the denture base can act as a shock absorber. Therefore, when there is a lack of interarch space, ceramic teeth are the most inappropriate choice as it is impossible to provide sufficient thickness for the resin base [7,15,23].

Matsuo and Matsuo [24] showed that fibroblasts started to respond to the pressure by increasing the intracellular calcium at a threshold level of 27 to 68 g/cm^2^ of pressure. Also, Berg et al [25] reported that in order to maintain the normal blood flow, continuous pressure applied to the denture-supporting tissues should not exceed 0.0013 MPa. The values obtained in the present study were higher than these values. Since pressure is the force applied per unit area, the small area of the samples’ base in this study (157 mm^2^) could be an explanation for the increased transmitted pressure. Therefore, the denture base should cover the maximum area within the physiologic limits [7,21].

Phunthikaphadr et al [7] measured pressure transmission under the denture base when a metal body applied force to acrylic baseplate resin, ceramic, and nanocomposite teeth. They reported the maximum and minimum pressure values with ceramic and nanocomposite teeth, respectively [7]. Using a metal body, a different measuring method, and a different loading system in the mentioned study led to a different conclusion from that of the present study. However, Arksornnukit et al [15] showed results similar to those of the present study, despite different loading systems.

One of the limitations of the present study was that the pressures beneath denture bases were evaluated in vitro using a unidirectional force. Therefore, future studies should be carried out with multidirectional loads to simulate the oral environment. Another limitation was that the model used in the present study consisted of only one denture tooth in a small resin block with no periosteum or bone. Furthermore, in the clinical situation, dentures have more base area compared to this model. Pressure transmission and distribution under maxillary and mandibular complete dentures may not be the same due to the different denture configuration.

## CONCLUSION

Within the limitations of the present study, acrylic teeth could be the most favorable choice to reduce the pressure beneath denture bases, especially when there is a lack of space or concern about bone resorption. In less sensitive situations, nanocomposite and composite-acrylic resin teeth may be used as alternatives.
